# Paeoniflorin Attenuated Oxidative Stress in Rat COPD Model Induced by Cigarette Smoke

**DOI:** 10.1155/2016/1698379

**Published:** 2016-11-28

**Authors:** Jinpei Lin, Fei Xu, Genfa Wang, Lingwen Kong, Qingli Luo, Yvbao Lv, Jiaqi Liu, Ying Wei, Lulu Li, Hongying Zhang, Jingcheng Dong

**Affiliations:** ^1^Department of Integrative Medicine, Huashan Hospital, Fudan University, 12 Middle Urumqi Road, Shanghai 200040, China; ^2^The Academy of Integrative Medicine of Fudan University, Shanghai 20040, China

## Abstract

Paeoniflorin (PF), a monoterpene glucoside, might have an effect on the oxidative stress. However, the mechanism is still unknown. In this study, we made the COPD model in Sprague-Dawley (SD) rats by exposing them to the smoke of 20 cigarettes for 1 hour/day and 6 days/week, for 12 weeks, 24 weeks, or 36 weeks. Our findings suggested that smoke inhalation can trigger the oxidative stress from the very beginning. A 24-week treatment of PF especially in the dosage of 40 mg/kg·d can attenuate oxygen stress by partially quenching reactive oxygen species (ROS) and upregulating antioxidant enzymes via an Nrf2-dependent mechanism.

## 1. Introduction

Chronic obstructive pulmonary disease (COPD) is the most common chronic respiratory condition in adults and is characterized by progressive airflow limitation that is not fully reversible [1]. Nowadays, cigarette smoke induced COPD is one of the most serious health problems in China. Oxidative stress is one of the most important features in the pathogenesis of COPD which aggravates chronic airway inflammation and remodeling. The enhanced oxidative stress in COPD patients results from increased amounts of reactive oxygen species (ROS) generated by various inflammatory, immune, and epithelial cells in the airways. Oxidative stress has important implications on the pathogenesis of COPD [2]. These include oxidative inactivation of antiproteases and surfactants, mucus hypersecretion, membrane lipid peroxidation, mitochondrial respiration, alveolar epithelial injury, remodeling of extracellular matrix, and apoptosis. An increased level of ROS is reflected by increasing the markers of oxidative stress in the airspaces, sputum, breath, lungs, and blood. A variety of oxidants, free radicals, and aldehydes are implicated in the pathogenesis of COPD. Antioxidant compounds may be of therapeutic value in monitoring the disease progression. Paeoniflorin (PF) is a monoterpene glucoside and the main component of the total glycosides of paeony (TGP) extracted from the roots of* Paeonia lactiflora*. PF is extensively studied for the utility in a number of therapeutic areas due to its anti-inflammatory, antioxidant, and anticancer properties [3–5]. In the present study, we investigated the protective role of PF against cigarette smoke mediated oxidative stress in rat model of COPD. Our results showed that PF treatment can restore the oxidant/antioxidant imbalance caused by cigarette smoke in lung tissues obtained from COPD rats.

## 2. Materials and Methods

### 2.1. Experimental Animals and Grouping

Eight-week-old male Sprague-Dawley (SD) rats (weighed 180–200 g) were purchased from S&P Shall Kay Laboratory Animal Co., Ltd. (Shanghai, China), and used in this study. Upon arrival, all animals were housed in polycarbonate cages under a 12-hour light-dark cycle with continuous access to food and water. The total of one hundred and forty-four male SD rats were randomly assigned to six groups (*n* = 24 per group, 4 per cage); each group has three stages (12 weeks; 24 weeks; 36 weeks): Normal Control group (NC group), COPD model group, Paeoniflorin low-dose group (12 mg/(kg·d)), Paeoniflorin middle-dose group (24 mg/(kg·d)), Paeoniflorin high-dose group (48 mg/(kg·d)), and Budesonide group (0.2 mg/(kg·d)) [6]. Animals were acclimated to the new housing environment for one week before tobacco smoke exposure began. All studies were performed in accordance with the recommendations of the Guide for the Care and Use of Laboratory Animals of Fudan University of Chinese Medicine and all procedures were performed under the supervision of the Animal Experimental Ethical Committee of Fudan University.

### 2.2. Tobacco Exposure

Groups of 120 SD rats each were exposed to tobacco smoke of 20 3R4F research cigarettes (Tobacco and Health Research Institute, University of Kentucky, KY) for 1 hour/day, 6 days/week, for either 12 weeks, 24 weeks, or 36 weeks, except for NC groups. A special smoke apparatus named inhalation tower (Inhalation Tower, Buxco, USA, ELS0113) for rat exposure to cigarette smoke was prepared. The inhalation tower comprising a plurality of tiers stackable one on top of another, and a plurality of trays for supporting more rats, wherein each tier has means for supplying gas to rats and for evacuating exhaled gas, wherein each tier has complementary formations formed on opposite ends thereof, where complementary formations are configured to enable stacked tiers to locate and engage with one another, and the complementary formations are also being configured at the periphery of the ends of the tiers so that one of the trays can be received between adjacent stacked tiers. Briefly, a group of rats were placed in the inhalation chamber. The cigarette smoke was consistently delivered into the chamber at a rate of approximately 15 min per cigarette and three cigarettes for each challenge. Rats were kept in the smoked chamber for 30 min in the morning and 30 min in the afternoon, 6 days a week, for 12, 24, and 36 weeks [7, 8]. After each exposure, the rats were returned to their cages. Control animals inhaled clean air only. The amount of cigarette was increased with gradient to reach target dose.

### 2.3. Drugs and Delivery

PF was purchased from Chengdu Must Bio-Technology Co., Ltd. (Chengdu, China). Paeoniflorin low-dose group (12 mg/(kg·d)), Paeoniflorin middle-dose group (24 mg/(kg·d)), and Paeoniflorin high-dose group (48 mg/(kg·d)) were intragastrically administered of different doses of PF (12, 24, and 48 mg·kg^−1^) per day for six days before being exposed to tobacco smoke. 0.9% of physiological saline Sulfasalazine at the same volume was used as a normal control drug, while the Budesonide (0.2 mg/(kg·d)), which was used in the long term management of COPD, was used as a positive control drug by using ultrasonic atomizing inhalation [9]. In addition, the COPD model group was intragastrically administered just the same as NC group.

### 2.4. Measurement of Pulmonary Function by Buxco's Research System

Pulmonary function was assessed directly by Buxco's Research System. All rats were anesthetized with 2% pentobarbital sodium (1 mL/100 g ip). Then a tracheotomy was made; the trachea was cannulated; the rats were placed in a whole-body plethysmography chamber (Buxco, USA) for anesthetized animals; the trachea was connected with the small animal ventilator. The spirometer special for small animals was applied to observe the following variables: the ratio of forced expiratory volume in the first 0.1 seconds (FEV0.1) and forced vital capacity (FVC) (FEV0.1/FVC%); maximal midexpiratory flow (MMEF); peak expiratory flow (PEF).

### 2.5. Immunohistochemistry

The middle lobe of the right lung was excised from each rat and fixed in 4% formalin. Sections 3 *μ*m thin from blocks were stained with hematoxylin and eosin stain (HE). Histopathological assessment (light microscopy) was performed blind on randomized sections. The severity of inflammatory cell infiltration in airway was estimated with a 4-point scoring system: the severity of peribronchial inflammation was graded semiquantitatively for the following features: 0, normal; 1, few cells; 2, a ring of inflammatory cells one cell layer deep; 3, a ring of inflammatory cells two to four cells deep; 4, a ring of inflammatory cells of more than four cells deep [10].

### 2.6. ELISA

Blood was collected and separated in a refrigerated centrifuge (at 3000–5000 rpm for 5 min) at 4°C. Serum was stored at −80°C until assays. Total antioxidant capacity (T-AOC), ROS, superoxide dismutase (SOD) activity, human hemeoxygenase 1 (HO-1), and malondialdehyde (MDA) were determined by T-AOC detection kit (F19119, Xitang, China), ROS detection kit (F18106, Xitang, China), total SOD assay kit (F16742, Xitang, China), HO-1 ELISA Kit (F15646, Xitang, China), and MDA ELISA Kit (F16194, Xitang, China), respectively, according to the manufacturer's instructions. All of the above kits were provided by Xitang (Shanghai, China).

### 2.7. Real-Time PCR

Total RNA was extracted from lung tissue using Trizol-Reagent (15596018, Invitrogen, Thermo Fisher Scientific, USA) according to the manufacturer's instructions (BioTNT, Shanghai, China). Reverse-transcription (RT) reactions were performed using iScriptTM cDNA Synthesis kit (A2010A202, BioTNT, BioTNT, China). The primers were used to detect activating transcriptional factor 4 (ATF4), nuclear factor erythroid 2 related factor-2 (Nrf2), SOD, HO-1, *γ*-glutamyl cysteine synthetase (*γ*-GCS), and *β*-actin as follows: *β*-actin: 5′ CCT CTA TGC CAA CAC AGT 3, 5′ AGC CAC CAA TCC ACA CAG 3; ATF-4: 5′ CTC GCC AAA GAG ATT CAG T, 5′ TAC AGC AAA CAC AGC ATC A; Nrf2: 5′ GAG GAT GGG AAA CCT TAC T, 5′ CTT CTT GCT CTT GGG AAC A; *γ*-GCS: 5′ CAA CAC AGT GGA GGA CAA C, 5′ TCT GGA AAG AAG AGG GAC T; SOD: 5′ CCA CGA GAA ACA AGA TGA C, 5′ GAC TCA GAC CAC ATA GGG A; HO-1: 5′ TTT CAG AAG GGT CAG GTG T, 5′ CTT GTT TCG CTC TAT CTC C. Quantitative RT-polymerase chain reactions (qRT-PCR) were performed by iQ SYBR Green Supermix of Bio-Rad. A negative control without cDNA template was run with every assay. Transcript copy number per subject was calculated by normalization to *β*-actin expression. The relative expression was calculated by the change-in-threshold (−ΔΔCT) method [11].

### 2.8. Western Blot Analysis

Lungs were homogenized in WB and IP lysis buffer (WB007, Xitang, China) and incubated for 30 min at 4°C. Proteins were extracted, respectively, according to instructions of a protein extraction kit (Xitang, China). Cell debris was removed by microcentrifugation, and supernatants were quickly frozen. The protein concentration was determined by Bradford assay (BSA) method. Proteins from the subcellular fractions were mixed with 2x SDS sample buffer (100 mmol L^−1^ Tris-HCl (pH 6.8), 4% SDS (w/v), 20% glycerol, 200 mmol L^−1^ DTT, and 0.1% bromophenol blue (w/v)) and boiled in a water bath for 5 min, before separation on 8% polyacrylamide gels for Nrf2 and *γ*-GCS, 12% polyacrylamide gels for GAPDH, ATF4 and HO-1, and 15% polyacrylamide gels only for SOD. After quantification by the BSA method, protein samples were separated by 10% SDS-PAGE for 4 h and subsequently transferred to nitrocellulose membranes. The filter was then blocked in tris-buffered saline containing 0.1% Tween-20 (TBST) and 5% dried milk powder (wt/vol) for 2 h at room temperature. The nitrocellulose filters were incubated with primary antibody (1 : 1000) (HO-1: AF3169, R&D Systems, USA; *γ*-GCS: AB190685, Abcam, USA; SOD: AF3787, R&D Systems, USA; Nrf2: MAB3925, R&D Systems, USA; ATF4: AB1371, Abcam, USA) or GAPDH (1 : 20000) (5G4-6C5, HyTest, Denmark) at 4 °C overnight. After washes with TBST, the filters were incubated with IgG HRP (1 : 10000) (115-035-003, Jackson ImmunoResearch, USA) for 1 h at room temperature and further washed for 30 min with TBST. Immunoreactive proteins were visualized using the enhanced chemiluminescence Western blotting detection system. Staining intensity of the bands was measured using a densitometer (Syngene, Braintree, UK) together with Genesnap and Genetools software (Syngene). To control sampling errors, the ratio of band intensities to GAPDH was obtained to quantify the relative protein expression level.

### 2.9. Statistical Analysis

Data was analyzed with the SPSS18.0 package. The results were presented as mean ± standard deviation. There were more than two groups; hence, we first used a normal testing method and homoscedasticity analyses via the Shapiro-Wilk method and the Levene method. If the data consisted of a normal distribution and homoscedasticity, we did one-factor analysis of variance (One-ANOVA). If the data were not consistent with a normal distribution, we did a rank test whose statistical significance was set at *P* < 0.01.

## 3. Results

### 3.1. PF Improved the Pulmonary Function in Rat COPD Model

**Figure 1:**
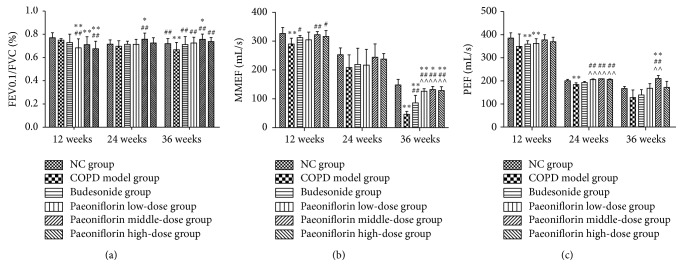
Effects of PF on pulmonary function of rats of different stages. The histograms of FEV0.1/FVC% (a), PEF (b), and MMEF (c), respectively, indicated successful formation of COPD. Data were mean ± SEM (*n* = 8). ^*∗*^ *P* < 0.05, ^*∗∗*^ *P* < 0.01, compared with the Normal Control group. ^#^ *P* < 0.05, ^##^ *P* < 0.01, compared with the COPD model group. ^∧∧^ *P* < 0.01, compared with Budesonide group.

In this experiment, cigarette smoke which is strongly linked to the pathogenesis of COPD was used to generate the rat COPD model. COPD rats showed a continuous decline in lung function parameters, like FEV0.1/FVC which is characterized for development of emphysema (Figure 1(a)). The airway enlargements as well as remodeling were reflected in the decrease of PEF and MMEF. Our results showed that PF treatment could improve FEV0.1/FVC%, PEF, and MMEF in time-dependent manner (Figures 1(a)–1(c)). Middle-dose of PF had a better effect than both the low-dose and high-dose (Figures 1(a)–1(c)). Compared with Budesonide, PF of all three dosages showed a more promising effect after treatment (Figures 1(a), 1(b), and 1(c)).

### 3.2. PF Improved Lung Histological Condition in Rat COPD Model

**Figure 2:**
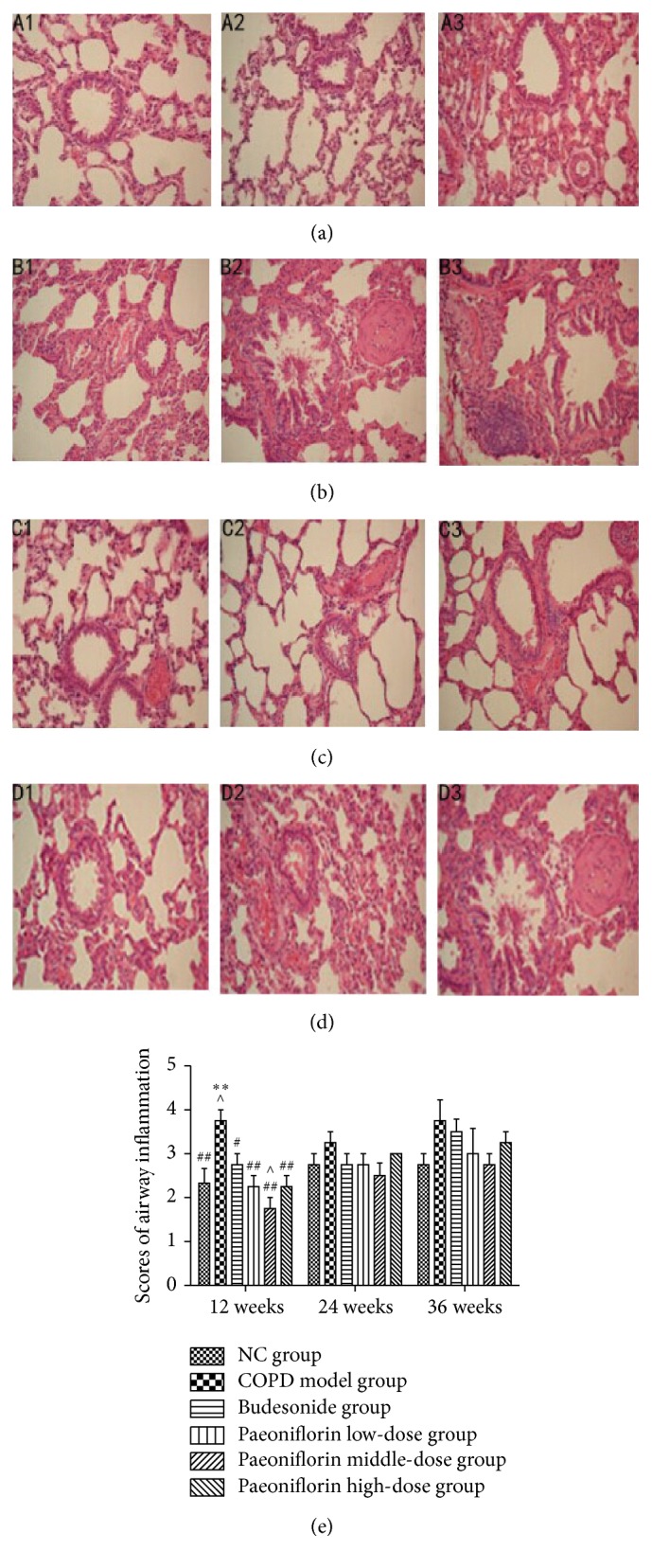
Histological analysis and airway inflammatory scores revealed typical pathological features. The right lung was removed for histopathologic examination using hematoxylin and eosin staining. (a) Normal Control (NC) group. (b) COPD model group. (c) Budesonide group. (d) Paeoniflorin middle-dose group. Original magnification, ×200. 1–3 presented the histology of lung at three stages, 12 weeks, 24 weeks, and 36 weeks, respectively. (e) The severity of airway inflammation scores. ^*∗∗*^ *P* < 0.01, compared with the Normal Control group. ^#^ *P* < 0.05, ^##^ *P* < 0.01, compared with the COPD group. ^∧^ *P* < 0.05, compared with Budesonide group.

We successfully used cigarette smoke to generate the COPD model in rats. The histological analysis of lung tissue showed an increased number of the infiltrated inflammatory cells in the COPD model group (Figure 2(b)) compared with NC group (Figure 2(a)) after 12 weeks of cigarette smoke exposure. PF treatment can help ameliorate the typical pathological features of COPD: for example, a decline of inflammatory cells around the bronchioles and mucus and the number of debris accumulated in the lumens of bronchioles (Figure 2(d)). Moreover, airway inflammation and edema were significantly reduced in the middle-dose group than any other groups including the Budesonide treated group (Figure 2(c)). Compared with other groups, airway inflammation was significantly more severe in the lungs of the COPD group. Administration of the middle dosage of PF resulted in a significant decrease of the lung inflammation score after 12 weeks compared with all other groups (Figure 2(e)).

### 3.3. PF Attenuated Oxidative Stress in COPD Model

**Figure 3:**
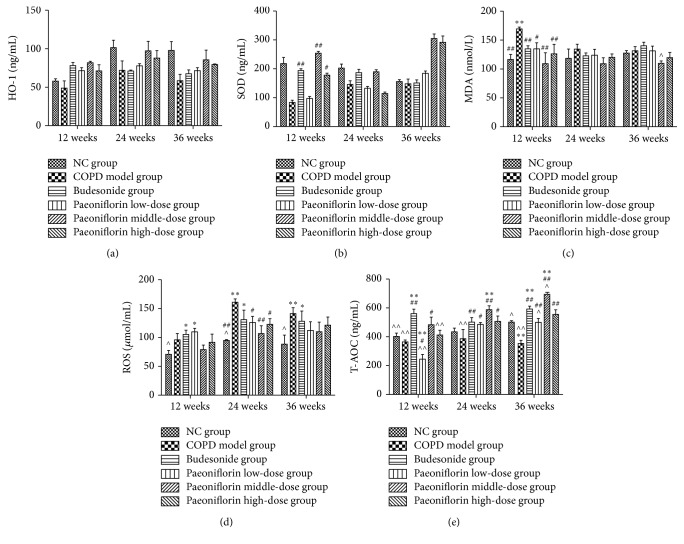
Levels of HO-1, SOD, MDA, ROS, and T-AOC in rat serum. In this study, we used Elisa to detect the level of oxidants like ROS (d) and MDA (c) as well as antioxidants including HO-1 (a), SOD (b), and T-AOC (e) in rat serum. ^*∗*^ *P* < 0.05, ^*∗∗*^ *P* < 0.01, compared with the Normal Control group. ^#^ *P* < 0.05, ^##^ *P* < 0.01, compared with the COPD group. ^∧^ *P* < 0.05, ^∧∧^ *P* < 0.01, compared with Budesonide group.

In our experiment, we confirmed the presence of a profound oxidant/antioxidant imbalance in the serum of rats with COPD. The oxidative stress reaches its peak at 24 weeks' inhalation of cigarette smoke. The level of specific markers of oxidant stress such as ROS was significantly increased after 24 weeks (Figure 3(d)). But for MDA, only in the first 12 weeks, it showed a significant increase compared with NC group (Figure 3(c)). In the meanwhile, the total antioxidation capacity (T-AOC) was remarkably increased to protect from cigarette smoke damage (Figure 3(e)). PF treatment can decreased ROS and MDA levels significantly in the early stage (12 weeks). It also showed a trend of increasing antioxidative biomarkers in the serum obtained from COPD rats (Figures 3(c) and 3(d)).

**Figure 4:**
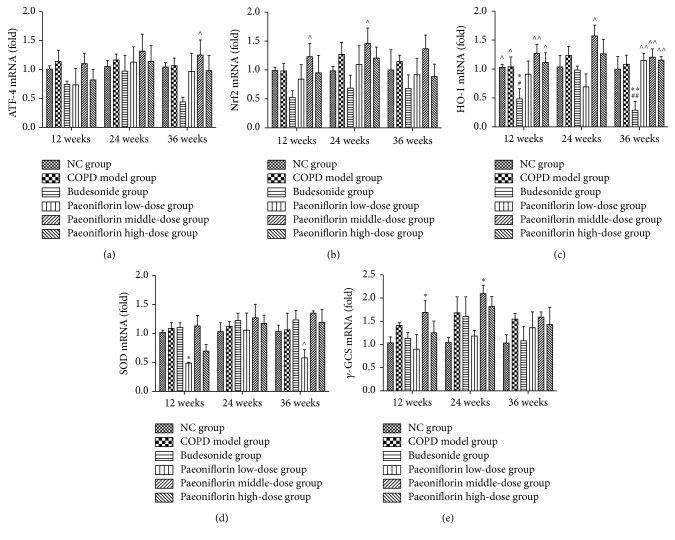
Regulation of mRNA expressions of antioxidant enzymes including HO-1, *γ*-GCS, and SOD via an Nrf2 dependent mechanism. PF of all three dosages significantly increased the total amount of mRNA expression of antioxidant enzymes by means of upregulating ATF-4 (a) and Nrf2 (b) mRNA expressions as well as HO-1 (c), SOD (d), and *γ*-GCS (e) in the lung tissue. ^*∗*^ *P* < 0.05, ^*∗∗*^ *P* < 0.01, compared with the Normal Control group. ^#^ *P* < 0.05, ^##^ *P* < 0.01, compared with the COPD group. ^∧^ *P* < 0.05, ^∧∧^ *P* < 0.01, compared with Budesonide group.

**Figure 5:**
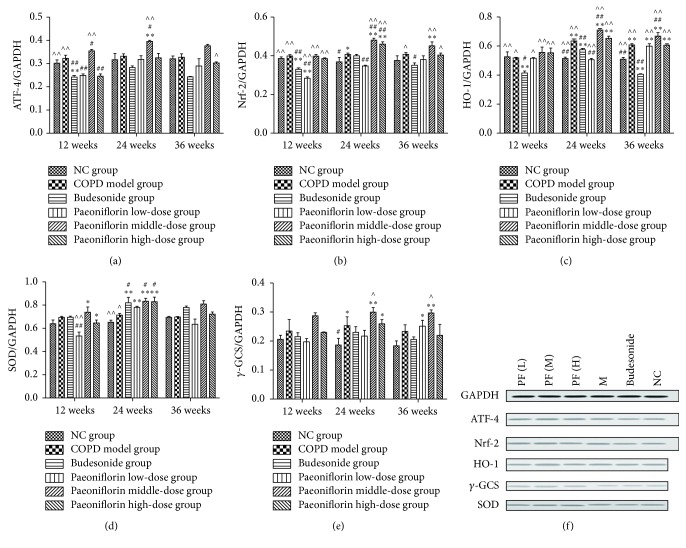
Estimate of protein expressions throughout Western blot. GAPDH was used as the loading control. The optical density for target protein is shown as a proportion of GAPDH optical density. Here we detected the total amount of protein expressions of ATF-4 (a), Nrf2 (b), HO-1 (c), SOD (d), and *γ*-GCS (e) in the lung tissue. ^*∗*^ *P* < 0.05, ^*∗∗*^ *P* < 0.01, compared with the Normal Control group. ^#^ *P* < 0.05, ^##^ *P* < 0.01, compared with the COPD group. ^∧^ *P* < 0.05, ^∧∧^ *P* < 0.01, compared with Budesonide group.

Cigarette smoke inhalation resulted in upregulation of mRNA expression of antioxidant enzymes such as HO-1, *γ*-GCS, and SOD (Figures 4(c)–4(e)). Moreover, Nrf2 and ATF4 mRNA expression was also increased in PF treated groups compared with Budesonide treatment (Figures 4(a) and 4(b)). Compared with all three stages, the transcription of antioxidant enzymes in the lung tissue was gradually increasing until 24 weeks of cigarette smoke inhalation. Later it was going down at the third period. Consistent with mRNA expression, PF of all three dosages significantly increased the total amount of Nrf2 and ATF4 proteins as well as *γ*-GCS, HO-1, and SOD proteins in the COPD lung tissue (Figures 5(a)–5(f)).

## 4. Discussion

COPD is the most common chronic respiratory condition in adults and has been considered as one of the most serious health problems in China [12]. Oxidative stress is one of the most important features in the pathogenesis of COPD which aggravates chronic airway inflammation and the remodeling of the extracellular matrix [13]. In this experiment, cigarette smoke which is strongly linked to the pathogenesis of COPD was used to generate the rat COPD model. Results from pulmonary function showed that lung function parameters of COPD model group were progressively decreasing, like FEV0.1/FVC, which is characterized for development of emphysema. PEF and MMEF suffer from a decline during the experiment. These two factors reflected airway enlargements as well as remodeling. But as we can see, the lung functions are also decreasing in the NC group of all three stages. That may be because lungs are continuously exposed to oxidants not only coming from air pollutants or cigarette smoke, but also being generated by metabolic reactions like mitochondrial electron transport during respiration or during activation of phagocytes. We do the experiment for totally 36 weeks; the rats also experience aging during the experiment. Moreover, these changes are in accordance with the histological analysis of lung tissue which presented that infiltrated inflammatory cells were significantly increased in the COPD model group compared with NC group. The scores of inflammation also support that airway inflammation and edema were more severe in the lungs of the COPD group compared with those groups containing different dosage of PF. Throughout the results of lung function and pathogenesis, we draw the conclusion that the rat model of COPD was successfully established by cigarette smoke.

Oxidative stress occurs when the balance between oxidants and antioxidants shifts in favor of oxidants [14, 15]. The results also suggested that cigarette smoke induced oxidative stress in the COPD model group by both the excess of oxidants and the depletion of antioxidants. Heme oxygenase-1, an enzyme degrading heme to carbon monoxide, iron, and biliverdin, has been considered to play a crucial role in cellular defense against stressful conditions [16]. Glutathione (GSH), a ubiquitous tripeptide thiol, is a vital intra- and extracellular protective antioxidant against oxidative/nitrosative stresses, which plays a key role in the control of proinflammatory processes in the lungs. Recent findings have suggested that GSH is important in immune modulation, remodeling of the extracellular matrix, apoptosis, and mitochondrial respiration. The rate-limiting enzyme in GSH synthesis is *γ*-glutamylcysteine synthetase (*γ*-GCS) [17]. Superoxide dismutases (SODs) are the only enzyme family with activity against superoxide radicals [18]. PF treatment decreased ROS and MDA levels, whereas it increased antioxidative biomarkers (T-AOC, activities of antioxidant enzymes SOD, HO-1, and *γ*-GCS) in lung tissues obtained from rats. But for MDA, we also observed a decrease in MDA concentration in serum of rat with COPD after 24 and 36 weeks when compared to 12 weeks. It might be because, in the first 12 weeks, the lung suffers from acute injury from cigarette smoke. So we speculated that the rats undergo an adaptive response to chronic oxidant exposure that ameliorates potential damage to lung cells from further oxidant stress. Consistent with our results, previous studies have reported that PF treatment can reduce the formation of ROS, the level of malondialdehyde (MDA), and lactate dehydrogenase (LDH) leakage and enhanced production of the endogenous antioxidants, glutathione (GSH), and superoxide dismutase (SOD) in EA.hy926 cells. The three dosages of PF treatment significantly induced HO-1 expression. Moreover, PF promoted the nuclear translocation of Nrf-2. The Paeoniflorin-induced HO-1 expression was abrogated by Nrf2 siRNA [19]. These results suggest that the protective effects of PF against COPD may be partly due to its potent antioxidant property.

Gene expression changes occur within the lung, probably as an initial defense mechanism but likely to become destructive after chronic tobacco smoke exposure. These changes are genes associated with oxidative stress, remodeling, and other pathways. Nrf2 is a redox-sensitive transcription factor that regulates the expression of phase II antioxidant genes and confers cytoprotection against oxidative stress [20, 21]. ATF4 is significantly upregulated in oxidative stress via the interaction of Nrf2. ATF4 may play a role in the transcriptional control of the target gene expression in the oxidative imbalance in COPD. There also appears to be an “unfolded” protein response in the COPD lung where proteins are upregulated in an attempt to control the oxidative onslaught of tobacco smoke. Activation of cytoprotective Nrf2/ATF4 genes can enhance inflammatory responses to oxidant insults.

Fortunately, the data shows that PF treatment can decrease the level of ROS production and resulted in significant ATF4 and Nrf2 expressions which are the main cause of increased level of antioxidant enzymes as compared with cigarette smoke exposure alone. The histograms of PEF, MMEF, and FEV0.1/FVC% show that lung function of COPD group was significantly decreasing compared with NC group. And the pulmonary function was gradually getting worse as time passes by. But there was no significant difference between NC group and Paeoniflorin middle-dose group, which means that a dosage of 40 mg/kg·d PF can protect lung function of rats from continuous cigarette smoke exposure. Moreover, PF treatment can also help ameliorate the typical pathological features of COPD throughout decreasing numerous inflammatory cells around the bronchioles, as well as the mucus and debris accumulated in the lumens of bronchioles. In our experiment, we also confirm the presence of a profound oxidant/antioxidant imbalance in the serum of rats with COPD which returns towards normal values during the course of PF treatment. The level of specific markers of oxidant stress like ROS was significantly increased. Meanwhile, the antioxidant cytokines were remarkably increasing to protect from cigarette smoke damage. In addition, the oxidative stress might reach its peak at 24 weeks' inhalation of cigarette smoke. Cigarette smoke inhalation resulted in upregulation of mRNA expressions of antioxidant enzymes including HO-1, *γ*-GCS, and SOD. PF of all three dosages significantly increased the total amount of mRNA expression of antioxidant enzymes by means of upregulating Nrf2 and ATF4 mRNA expressions. Compared with all three stages, the transcription in the lung tissue was gradually increasing until 24 weeks of cigarette smoke inhalation. Later it was going down at the third period. Consistent with mRNA expression, PF of all three dosages significantly increased the total amount of Nrf2 and ATF4 protein expression as well as *γ*-GCS, HO-1, and SOD protein expression in the lung tissue.

Therefore, targeting oxidative stress with antioxidants or boosting the endogenous levels of antioxidants is likely to be beneficial in the treatment of COPD. As we all know, lung has an efficient but complicated antioxidant defense system, where antioxidant enzymes and related detoxification enzymes have a cell specific expression and compartmentalization and variable induction capacities. Cigarette smoke leads to an induction of some of these enzymes, especially in the bronchial and alveolar epithelium, possibly reflecting high oxidant burden in these locations. All the enzymes mentioned above are of great importance in COPD model. The experimental findings suggested that PF attenuates cigarette smoke induced oxidative stress not only by quenching ROS but also by upregulating HO-1 *γ*-GCS SOD via an Nrf2 dependent mechanism.

We still have a lot more to work on if we want to apply PF treatment into clinic use. From this experiment, we can not specify the very targeting spot. But result from our experiment still showed a promising future of PF treatment by finally improving the lung function as well as ameliorating the recovery from the oxidant/antioxidant imbalance. At least we figure out that the effect of PF might be related to upregulating HO-1 *γ*-GCS SOD via an Nrf2 dependent mechanism.

## 5. Conclusion

Cigarette smoke inhalation can cause the oxidant/antioxidant imbalance in lung tissues starting from the very beginning. In cigarette smoke induced rat model, the oxidant burden is further increased for a number of reasons. Oxidative stress, resulting from the increased oxidative burden and decreased level of antioxidant proteins, plays a role in the pathophysiology of smoking-related pulmonary emphysema. Antioxidant enzyme may be compensatory upregulated against oxidative stress in COPD. ATF4 enhance transcription Nrf2 and upregulate the expression of the downstream target genes such as *γ*-GCS by forming heterodimers with Nrf2. The sooner PF treatment is started especially in the dosage of 40 mg/kg·d, the better outcome it will be at the end of the experiment throughout quenching ROS and upregulating antioxidant enzymes like HO-1, *γ*-GCS, and SOD via an Nrf2 dependent mechanism.
